# Exploring sex differences for acute ischemic stroke clinical, imaging and thrombus characteristics in the INTERRSeCT study

**DOI:** 10.1177/0271678X231189908

**Published:** 2023-07-17

**Authors:** Alexander D Rebchuk, Michael D Hill, Mayank Goyal, Andrew Demchuk, Shelagh B Coutts, Negar Asdaghi, Dar Dowlatshahi, Jessalyn K Holodinsky, Enrico Fainardi, Jai Shankar, Mohamed Najm, Marta Rubiera, Alexander V Khaw, Wu Qiu, Bijoy K Menon, Thalia S Field

**Affiliations:** 1 Division of Neurosurgery, University of British Columbia, Vancouver, BC, Canada; 2 Department of Clinical Neurosciences, 2129 Cumming School of Medicine, University of Calgary, Calgary, AB, Canada; 3 Calgary Stroke Program, University of Calgary, Calgary, AB, Canada; 4 Department of Radiology, University of Calgary, Calgary, AB, Canada; 5 Department of Neurology, University of Miami Miller School of Medicine, Miami, FL, USA; 6 School of Epidemiology and Public Health, Faculty of Medicine, University of Ottawa, Ottawa, ON, Canada; 7 Department of Medicine (Neurology), University of Ottawa, Ottawa Hospital Research Institute, Ottawa, ON, Canada; 8 Department of Experimental and Clinical Biomedical Sciences, University of Florence, Florence, Italy; 9 Department of Radiology, 8664 University of Manitoba, Winnipeg, MB, Canada; 10 Neurology Department, Hospital Vall d’Hebron, Barcelona, Spain; 11 Department of Clinical Neurosciences, University of Western Ontario, London, ON, Canada; 12 Division of Neurology, University of British Columbia, Vancouver, BC, Canada; 13 Djavad Mowafaghian Centre for Brain Health, University of British Columbia, Vancouver, BC, Canada; 14 Vancouver Stroke Program, Vancouver, BC, Canada

**Keywords:** Stroke, menopause, collaterals, thrombus, recanalization

## Abstract

Women, especially following menopause, are known to have worse outcomes following acute ischemic stroke. One primary postulated biological mechanism for worse outcomes in older women is a reduction in the vasculoprotective effects of estrogen. Using the INTERRseCT cohort, a multicentre international observational cohort studying recanalization in acute ischemic stroke, we explored the effects of sex, and modifying effects of age, on neuroradiological predictors of recanalization including robustness of leptomeningeal collaterals, thrombus burden and thrombus permeability. Ordinal regression analyses were used to examine the relationship between sex and each of the neuroradiological markers. Further, we explored both multiplicative and additive interactions between age and sex. All patients (n = 575) from INTERRseCT were included. Mean age was 70.2 years (SD: 13.1) and 48.5% were women. In the unadjusted model, female sex was associated with better collaterals (OR 1.37, 95% CIs: 1.01–1.85), however this relationship was not significant after adjusting for age and relevant comorbidities. There were no significant interactions between age and sex. In a large prospective international cohort, we found no association between sex and radiological predictors of recanalization including leptomeningeal collaterals, thrombus permeability and thrombus burden.

## Background

Multiple prospective studies have shown an association between sex and ischemic stroke severity, with females having worse outcomes than males.^1–3^ Stroke risk also increases markedly with age in women, doubling within the 10 years following menopause.^4^ The shift in risk profile and prognosis after stroke in post-menopausal women is thought to be due in part to the loss of the vasculoprotective effects of sex steroid hormones, particularly estrogen. Estrogen interacts with cerebral endothelium and thus may have an effect on collateral circulation. Additionally, estrogen suppresses endothelial plasminogen activator inhibitor and influences the levels of several clotting factors.^5^ Thus, there may be differences in clot burden and thrombus composition that may also affect likelihood of recanalization in pre-menopausal versus post-menopausal women.

In INTERRseCT, a multicentre international observational study studying recanalization in ischemic stroke, clot burden score and thrombus permeability were both independently associated with recanalization in both patients treated with thrombolysis and those treated conservatively.^6^ Robust collateral circulation is known to predict more favorable outcomes after stroke.^6^ In the interest of exploring potential biological factors impacting disparities in stroke outcome between men and women, we explored the effects of sex, and modifying effects of age, on these predictors of recanalization and more favourable outcome.

## Methods

### Study design

The details of the INTERRSeCT protocol were published previously.^6^ The experimental protocol was approved by the Conjoint Health Research Ethics Board at the University of Calgary and conformed to the Declaration of Helsinki. All participants provided written informed consent for the study. Briefly, the study enrolled patients with acute ischemic stroke and intracranial occlusion on baseline CT angiogram (CTA).^6^ Eligibility criteria included patients presenting to an emergency department within 12 hours of last known well and older than 40 years. Patients with vertebrobasilar artery occlusion were excluded. The study included patients that did and did not receive intravenous (IV) thrombolysis.

Demographic data, including relevant past medical history, smoking status, medication use on admission, including exogenous hormones, were recorded. Patients underwent a baseline head and neck CTA. An imaging expert blinded to all clinical information read all images using OsiriX version 3.5. Extent of intracranial thrombus burden was measured with the clot burden score. A complete occlusion of the ipsilateral anterior circulation vessels has a clot burden score of 0; no occlusion has a score of 10.^6,7^ Thrombus permeability was assessed using residual flow grades as follows: grade 0, no contrast permeation of thrombus; grade 1, contrast permeating diffusely through thrombus; grade 2, tiny hairline lumen or streak of well-defined contrast within the thrombus extending either through its entire length or part of thrombus.^6^ Collaterals (anterior cerebral-middle cerebral artery and posterior cerebral artery-middle cerebral artery) were scored from 0–10 using a previously published methodology, where a higher score indicates better collateral flow.^6^

### Statistical analysis

This was a post-hoc analysis. As data on menopausal status was not collected, we used age of 50 years as a proxy for menopausal status. We selected age 50 years as a proxy based on the population-level Canadian Longitudinal Study on Aging, in which the median self-reported age of natural menopause was 51 (IQR 49–54),^8^ and a global meta-analysis citing the mean age of natural menopause as 48.8 (95% CI 48.3–49.2).^8,9^ We performed sensitivity analyses using additional cutoff values (55 years and 60 years) as proxies for menopause. Cut-points lower than age 50 were not explored due to the small number of female participants under age 50.

We used univariable and multivariable ordinal logistic regression to examine the relationship between sex and each of collateral status, thrombus permeability and clot burden scores, and looked for multiplicative and additive interactions between age and sex. Age was explored both as a continuous variable and with dichotomous age cut-points (≤50, ≤55 and ≤60), serving as proxies for menopausal status. Variables were included in the multivariable model if they met a threshold of *p < *0.1 in univariate analysis, or were forced into multivariable modeling if considered clinically relevant. We repeated our ordinal regression analyses, restricted to female patients, to examine relationships between dichotomous age cut-points and each of collateral status, thrombus permeability and clot burden. We further explored the effect of age and sex on our dependent variables in our logistic regression model by examining for multiplicative interactions with an age*sex term, and by using an additive interaction model. To explore additive interaction we created dummy variables for age (<50, 50–59, >60 years) and sex (female and male). We compared clinical outcomes, specifically modified Rankin scale (mRS) and NIH Stroke Scale (NIHSS) scores at 90 days, using the Mann-Whitney U test. All data analyses were conducted in SPSS Statistics (version 26.0, IBM Corp., Armonk, NY). Significance was set at *p < *0.05. Given the hypothesis-generating nature of our study, no corrections were made for multiple comparisons.

## Results

**Figure 1.**
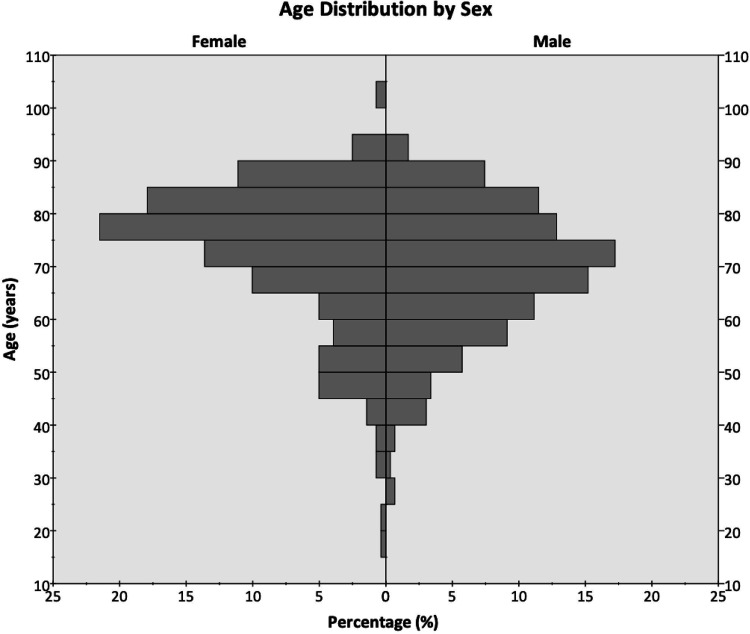
Distribution of age by sex in the INTERRseCT cohort.

Our analyses included all patients (n = 575) from INTERRseCT. Women comprised nearly half (48.5%) of the cohort. Mean age was 70.2 years (SD: 13.1). Distribution of age by sex is summarized in Figure 1. Proportion of participants aged ≤50, ≤55 and ≤60 years were 8.0% (8.6% of female patients), 15.0% (14.3% of females) and 21.2% (18.3% of females), respectively.

Overall, women were 6 years older than men (*p < *0.001), with a higher proportion of hypertension (8% difference, *p = *0.035), less coronary artery disease (7% difference, *p = *0.034), less dyslipidemia (12% difference, *p = *0.003), and fewer with a smoking history (25% difference, *p < *0.001) compared to men (Table 1). Three women were taking an oral contraceptive agent at the time of their event, five were taking hormone replacement therapy, and one was taking a selective estrogen receptor modulator (raloxifene). There was no difference in baseline antiplatelet or anticoagulant use between sexes. Baseline clinical characteristics are summarized in Table 1.

**Table 1.** Baseline characteristics of male and female participants.

Baseline demographics	Male (n = 296)	Female (n = 279)	Significance (*p*)
Mean age (median, IQR)	70 (61, 78)	76 (65, 82)	<0.001*
Hypertension (n, %)	171 (58)	185 (66)	0.035*
Dyslipidemia (n, %)	114 (39)	75 (27)	0.003*
Ever Smoker (n, %)	168 (58)	65 (23)	<0.001*
Diabetes type 2 (n, %)	50 (20)	26 (9)	0.18
Atrial fibrillation (n, %)	89 (30)	88 (32)	0.702
Coronary artery disease (n, %)	71 (24)	47 (17)	0.034*
Congestive heart failure (n, %)	10 (3)	9 (3)	0.919
Malignancy (n, %)	11 (4)	14 (5)	0.444
Antiplatelet use (n, %)	112 (38)	89 (32)	0.136
Anticoagulant use (n, %)	35 (12)	40 (14)	0.371
Baseline NIHSS (median, IQR)	14 (8, 19)	15 (9, 19)	0.167

Asterisk (*) indicates significance at p < 0.05.mRS: modified Rankin Scale; NIHSS: National Institutes of Health Stroke Scale.

In unadjusted analysis, female sex was associated with better collaterals (OR 1.37, 95% CIs: 1.01–1.85, *p* = 0.04). However, this relationship was not significant after adjusting for age and relevant comorbidities. We found no association between sex and clot burden score, or thrombus permeability (Table 2). Our results did not change when exploring different age cut-points for female participants (Table 3).

**Table 2.** Unadjusted ordinal regression analysis exploring association between sex, age and comorbidities, and baseline radiological predictors of recanalization (collaterals, thrombus permeability and clot burden score).

	Improved collaterals	Higher thrombus permeability	Lower clot burden
Variable	OR (95% Cis)	OR (95% Cis)	OR (95% Cis)
Sex (female)	1.37 (1.01–1.85)*	0.83 (0.56–1.27)	1.03 (0.76–1.39)
Age (continuous)	1.00 (0.98–1.01)	0.99 (0.97–1.00)	1.00 (0.99–1.01)
Age ≤50 years	1.08 (0.62–1.88)	0.98 (0.46–2.08)	1.20 (0.68–2.09)
Age ≤55 years	1.06 (0.70–1.62)	1.14 (0.65–2.00)	1.09 (0.71–1.68)
Age ≤60 years	1.10 (0.76–1.59)	1.43 (0.89–2.30)	0.95 (0.66–1.37)
Hypertension	0.88 (0.64–1.21)	0.72 (0.48–1.09)	1.25 (0.92–1.71)
Dyslipidemia	0.78 (0.56–1.08)	1.24 (0.81–1.90)	1.25 (0.90–1.73)
Atrial fibrillation	0.59 (0.42–0.83)*	0.68 (0.42–1.08)	0.80 (0.58–1.10)
Diabetes	1.07 (0.67–1.70)	0.70 (0.37–1.31)	1.44 (0.92–2.25)
Smoking	0.83 (0.61–1.14)	1.13 (1.00–1.67)	0.83 (0.60–1.13)
Coronary Artery Disease	0.67 (0.45–0.98)*	1.09 (0.66–1.79)	0.86 (0.59–1.24)
Congestive Heart Failure	0.48 (0.20–1.17)	0.47 (0.11–2.08)	0.64 (0.28–1.48)
Cancer	0.94 (0.46–1.93)	1.85 (0.80–4.31)	0.90 (0.45–1.82)
Antiplatelet use	0.72 (0.52–0.99)*	0.69 (0.44–1.08)	0.79 (0.57–1.09)
Anticoagulant use	0.69 (0.44–1.01)	1.02 (0.56–1.86)	0.85 (0.55–1.31)

For collaterals, OR indicate likelihood of *improved* collateral flow. For thrombus permeability, OR indicates likelihood of *higher* permeability. For clot burden, OR indicates likelihood of *lower* clot burden. Asterisk (*) indicates significance at *p < *0.05.

**Table 3.** Unadjusted ordinal regression models, restricted to female patients, exploring associations between dichotomous age cut-points and collaterals, thrombus permeability and clot burden score.

	Age		
	≤50 years	≤55 years	≤60 years
	OR (95% CIs)	OR (95% CIs)	OR (95% CIs)
Collaterals	1.15 (0.53–2.46)	1.04 (0.57–1.90)	0.92 (0.53–1.58)
Thrombus Permeability	0.67 (0.19–2.36)	1.45 (0.64–3.26)	1.55 (0.75–3.20)
Clot Burden Score	1.36 (0.61–3.06)	1.05 (0.57–1.92)	0.86 (0.50–1.47)

For collaterals, ORs indicate likelihood of *improved* collateral flow; for thrombus permeability, ORs indicate likelihood of *higher* permeability; for clot burden, ORs indicate likelihood of *lower* clot burden.

There were no significant multiplicative or additive interactions between age and sex when we used dummy variables for age (i.e. <50, 50–59, >60 years). In our multiplicative model, there were no significant age*sex interaction for collaterals (OR 0.88 [95% CI: 0.53–1.45]), thrombus permeability (OR 0.86 [95% CI: 0.44–1.65]) or clot burden (OR 0.93 [95% CI: 0.56–1.54]). The additive interactions from this model also did not find any significant effects of sex on the relationship between age and collaterals (OR 0.97 [95% CI: 0.24–3.85]), age and thrombus permeability (OR 0.56 [95% CI: 0.09–3.45]) or age and clot burden (OR 0.85 [95% CI: 0.21–3.45]). Similarly, there were no significant additive effects of age on the relationship between sex and collaterals (OR 1.03 [95% CI: 0.72–1.46]), sex and thrombus permeability (OR 0.93 [95% CI: 0.57–1.53]), or sex and clot burden (OR 1.01 [95% CI: 0.70–1.45]).

In unadjusted models, testing for the proportional odds assumption was satisfied (i.e. *p > *0.05) for all variables except for hypertension and collaterals (*p = *0.018), diabetes and collaterals (*p < *0.001), and diabetes and clot burden (*p < *0.001). In unadjusted ordinal regression models restricted to female patients, testing for the proportional odds assumption was satisfied (i.e. *p > *0.05) for all variables.

Workflow-related factors, such as onset-to-CT and CT-to-thrombolysis did not significantly differ by sex. However, females were observed to have a 12-minute faster CT-to-groin-puncture time compared to males (Table 4). There were no differences by sex with respect to rates of either thrombolysis or endovascular therapy.

**Table 4.** Workflow times and treatment strategy comparison between male and female participants.

	Male (n = 296)	Female (n = 279)	Significance (*p*)
Workflow times
Onset-to-CT (minutes; median, IQR)	115 (72, 171)	114 (75, 196)	0.169
CT-to-thrombolysis (minutes; median, IQR)	24 (17, 32)	23 (18, 36)	0.383
CT-to-groin-puncture (minutes; median, IQR)	74 (52, 98)	62 (42, 80)	0.001*
Treatment			
Thrombolysis alone (n, %)	137 (46)	138 (49)	0.46
Thrombolysis + EVT (n, %)	24 (8)	24 (9)	
EVT alone (n, %)	100 (34)	95 (34)	
Conservative (n, %)	35 (12)	22 (8)	

Time is given in minutes. Asterisk (*) indicates significance at *p < *0.05.

At 90 days post-stroke there were no significant differences between sexes for mRS or NIHSS outcomes. Median (IQR) 90-day mRS for males was 2 (1–4) and 2 (1–4) for females, *p = *0.1. Median (IQR) 90-day NIHSS for males was 2 (0–8) and 2 (0–13) for females, *p = *0.69. There were no significant age*sex interactions for either mRS or NIHSS outcomes.

## Discussion

In an international cohort of acute ischemic stroke patients, we found no association between sex and radiographic prognostic markers of recanalization. In our unadjusted model, female sex was marginally associated with better collaterals, but this effect was confounded as it disappeared after including relevant co-variables in our adjusted model. We found no evidence to support the hypothesis that cerebrovascular collaterals or thrombus characteristics were modified by the effect of pre- versus post-menopausal age and, indirectly, found no suggestion that these factors may contribute to sex differences in age-related post-stroke outcomes.

We observed no difference in collaterals between women of pre- and post-menopausal age with acute ischemic stroke. Based on evidence predominantly from rodent models, estrogen is thought to be protective in ischemic stroke via vasodilatory and anti-inflammatory mechanisms.^10–14^ Additionally, in a small study in healthy volunteers, premenopausal women demonstrated better cerebrovascular reactivity compared to post-menopausal women and age-matched men.^15^ Nonetheless, our findings are congruent with other clinical studies that have failed to find an association between sex and collateral status.^16–19^

In our cohort, sex did not meaningfully contribute to radiographic variability in collaterals in ischemic stroke patients. We have previously found in this cohort that 45–53% of between-patient variability in leptomeningeal collaterals is explained by patient demographics, comorbidities, clinical and laboratory variables.^20^ Genetic factors, including ApoE genotype, were not explored and may also contribute to between-patient variability in leptomeningeal collaterals, and may interact with sex in cerebrovascular regulation.^21^ Sex differences in gene expression provides a hypothesized mechanism for sexual dimorphism in microvascular function and responsive to ischemic events.^22^ Rodent models of cerebral blood flow have shown that sex differences in expression of mitochondrial proteins in both cerebral micro-vessels and large vessels lead to differences in mitochondrial-mediated dilation following ischemia.^23,24^ We suspect that any effect of sex on collaterals in our sample was attenuated when other confounding factors affecting collateral status were considered.

We also found no relationship between thrombus permeability or clot burden score and women of pre- and post- menopausal age. Estrogen is known to influence the clotting cascade, and exogenous estrogen is associated with increased risk of both venous and arterial thromboembolic events.^25^ However, it is uncertain as to whether estrogen would mediate differences in the histopathology of an arterial thrombus, and whether these differences, if present, would be reflected radiographically. It is unclear whether our findings signify a true absence of a relationship between pre- versus post-menopausal age and these thrombus characteristics, or whether our data are underpowered to detect a relationship. Whether endogenous estrogen levels are associated with different thrombus characteristics warrants further study, as this information could inform future personalized strategies for reperfusion.

Previous studies in Canada and elsewhere have described longer treatment times for women than men with acute ischemic stroke, and lower rates of reperfusion therapy being administered to women.^26–28^ Contrary to these previously published data, in our cohort, rates of reperfusion therapy (both tPA and/or EVT) were similar between and sexes and there were no workflow-related differences by sex apart from a faster CT-to-groin puncture time in females. Further, despite the older age of females in our cohort, the 90-day mRS and NIHSS outcomes were similar to male patients, and there was no significant interaction effect between sex and age with regards to clinical outcomes at 90 days post stroke. It is reassuring that in this study cohort the workflow times, treatment cohorts and outcomes are similar, suggesting that women appear to be getting appropriate care for acute stroke and benefiting as much as men. These findings may be reflective of local patterns of practice at study sites, which were at academic hospitals. Alternatively, there could be biases inherent to the INTERRSeCT cohort that attenuate sex differences that may be occurring in routine clinical practice, though this is less likely in the context of consecutive recruitment.

This study provides novel preliminary data exploring the relationship between sex and radiographic predictors of recanalization, and the potential modifying effects of age on this relationship. Our work was intended to be hypothesis-generating, and has several limitations. As expected, in keeping with the demographics of ischemic stroke we had a small number of female participants of pre-menopausal age. Our neutral results may therefore be due in part to Type II error. Similarly, small numbers prevent us from examining any potential mediating effect of exogenous hormone or estrogen receptor agonist use. Another limitation is the lack of primary data collected on menopausal status. By using age as a proxy for menopausal status, we may have misclassified pre- or post-menopausal women. Future work examining stroke-related outcomes should seek to prospectively collect data on menopausal status.

## Conclusions

In an international cohort of acute ischemic stroke patients presenting with intracranial occlusions on CTA we found no association between sex and radiological predictors of thrombus recanalization. More work is warranted to better elucidate genetic, biologic and social contributions to sex and gender differences in stroke risk and prognosis after stroke.

## References

[1] Appelros P Stegmayr B Terént A. Sex differences in stroke epidemiology: a systematic review. Stroke 2009; 40: 1082–1090. 19211488 10.1161/STROKEAHA.108.540781

[bibr2-0271678X231189908] Phan HT Blizzard CL Reeves MJ , et al. Sex differences in long-term mortality after stroke in the INSTRUCT (INternational STRoke oUtComes sTudy): a meta-analysis of individual participant data. Circ Cardiovasc Qual Outcomes 2016; 47: e003436. 10.1161/CIRCOUTCOMES.116.003436 28228454

[3] Joundi RA Adekanye J Leung AA , et al. Health state utility values in people with stroke: a systematic review and meta-analysis. J Am Heart Assoc 2022; 11: e024296. 35730598 10.1161/JAHA.121.024296 PMC9333363

[4] Benkhadra K Mohammed K Al Nofal A , et al. Menopausal hormone therapy and mortality: a systematic review and meta-analysis. J Clin Endocrinol Metab 2015; 100: 4021–4028. 26544652 10.1210/jc.2015-2238

[5] RoyO’Reilly M McCullough LD. Sex differences in stroke: the contribution of coagulation. Exp Neurol 2014; 259: 16–27. 24560819 10.1016/j.expneurol.2014.02.011 PMC4127336

[6] Menon BK Al-Ajlan FS Najm M , et al. Association of clinical, imaging, and thrombus characteristics with recanalization of visible intracranial occlusion in patients with acute ischemic stroke. JAMA 2018; 320: 1017–1026. 30208455 10.1001/jama.2018.12498 PMC6143104

[7] Goyal M Menon BK Krings T , et al. What constitutes the M1 segment of the middle cerebral artery? J Neurointerv Surg 2016; 8: 1273–1277. 26863104 10.1136/neurintsurg-2015-012191

[8] Costanian C McCague H Tamim H. Age at natural menopause and its associated factors in Canada: cross-sectional analyses from the Canadian Longitudinal Study on Aging. Menopause 2018; 25: 265–272. 28968303 10.1097/GME.0000000000000990

[9] Schoenaker DAJM Jackson CA Rowlands JV , et al. Socioeconomic position, lifestyle factors and age at natural menopause: a systematic review and meta-analyses of studies across six continents. Int J Epidemiol 2014; 43: 1542–1562. 24771324 10.1093/ije/dyu094 PMC4190515

[10] Krause DN Duckles SP Pelligrino DA. Influence of sex steroid hormones on cerebrovascular function. J Appl Physiol (1985) 2006; 101: 1252–1261. 16794020 10.1152/japplphysiol.01095.2005

[bibr11-0271678X231189908] Faber JE Moore SM Lucitti JL , et al. Sex differences in the cerebral collateral circulation. Transl Stroke Res 2017; 8: 273–283. 27844273 10.1007/s12975-016-0508-0 PMC5429998

[bibr12-0271678X231189908] Liu F McCullough LD. Interactions between age, sex, and hormones in experimental ischemic stroke. Neurochem Int 2012; 61: 1255–1265. 23068990 10.1016/j.neuint.2012.10.003 PMC3516397

[bibr13-0271678X231189908] Alkayed NJ Harukuni I Kimes AS , et al. Gender-linked brain injury in experimental stroke. Stroke 1998; 29: 159–165; discussion 166. 10.1161/01.str.29.1.159 9445346

[14] Pelligrino DA Santizo R Baughman VL , et al. Cerebral vasodilating capacity during forebrain ischemia: effects of chronic estrogen depletion and repletion and the role of neuronal nitric oxide synthase. Neuroreport 1998; 9: 3285–3291. 9831465 10.1097/00001756-199810050-00026

[15] Matteis M Troisi E Monaldo BC , et al. Age and sex differences in cerebral hemodynamics: a transcranial doppler study. Stroke 1998; 29: 963–967. 9596243 10.1161/01.str.29.5.963

[16] Nannoni S Sirimarco G Cereda CW , et al. Determining factors of better leptomeningeal collaterals: a study of 857 consecutive acute ischemic stroke patients. J Neurol 2019; 266: 582–588. 30610425 10.1007/s00415-018-09170-3

[bibr17-0271678X231189908] Malik N Hou Q Vagal A , et al. Demographic and clinical predictors of leptomeningeal collaterals in stroke patients. J Stroke Cerebrovasc Dis 2014; 23: 2018–2022. 25088172 10.1016/j.jstrokecerebrovasdis.2014.02.018

[bibr18-0271678X231189908] Menon BK Smith EE Coutts SB , et al. Leptomeningeal collaterals are associated with modifiable metabolic risk factors. Ann Neurol 2013; 74: 241–248. 23536377 10.1002/ana.23906 PMC3836863

[19] Liebeskind DS Tomsick TA Foster LD , et al. Collaterals at angiography and outcomes in the interventional management of stroke (IMS) III trial. Stroke 2014; 45: 759–764. 24473178 10.1161/STROKEAHA.113.004072 PMC3977615

[20] Rebchuk AD Field TS Hill MD , et al. Determinants of leptomeningeal collateral status variability in ischemic stroke patients. Can J Neurol Sci 2022; 49: 767–773. 34585652 10.1017/cjn.2021.226

[21] Wang R Oh JM Motovylyak A , et al. Impact of sex and APOE ε4 on age-related cerebral perfusion trajectories in cognitively asymptomatic Middle-aged and older adults: a longitudinal study. J Cereb Blood Flow Metab 2021; 41: 3016–3027. 34102919 10.1177/0271678X211021313 PMC8545048

[22] Chandra PK Cikic S Baddoo MC , et al. Transcriptome analysis reveals sexual disparities in gene expression in rat brain microvessels. J Cereb Blood Flow Metab 2021; 41: 2311–2328. 33715494 10.1177/0271678X21999553 PMC8392780

[23] Cikic S Chandra PK Harman JC , et al. Sexual differences in mitochondrial and related proteins in rat cerebral microvessels: a proteomic approach. J Cereb Blood Flow Metab 2021; 41: 397–412. 32241204 10.1177/0271678X20915127 PMC8370005

[24] Rutkai I Dutta S Katakam PV , et al. Dynamics of enhanced mitochondrial respiration in female compared with male rat cerebral arteries. Am J Physiol Heart Circ Physiol 2015; 309: H1490–500. 26276815 10.1152/ajpheart.00231.2015 PMC4666975

[25] Lisabeth L Bushnell C. Stroke risk in women: the role of menopause and hormone therapy. Lancet Neurol 2012; 11: 82–91. 22172623 10.1016/S1474-4422(11)70269-1 PMC3615462

[26] Uchida K Yoshimura S Sakai N , et al. Sex differences in management and outcomes of acute ischemic stroke with large vessel occlusion. Stroke 2019; 50: 1915–1918. 31167622 10.1161/STROKEAHA.119.025344

[bibr27-0271678X231189908] Persky RW Turtzo LC McCullough LD. Stroke in women: disparities and outcomes. Curr Cardiol Rep 2010; 12: 6–13. 20425178 10.1007/s11886-009-0080-2 PMC2861793

[28] Cordonnier C Sprigg N Sandset EC , et al. Stroke in women – from evidence to inequalities. Nat Rev Neurol 2017; 13: 521–532. 28731036 10.1038/nrneurol.2017.95

